# Maternity and Child-Welfare Centres

**Published:** 1921-07-09

**Authors:** 


### THE PUBLIC WELL-BEING.

### Maternity and Child-Welfare Centres.

### HOW TO IMPROVE THEIR WORK AND INFLUENCE.

Baby Week appropriately sees the issue of a valuable report on Maternity and Child-Welfare Work in relation to the Reduction of Infant Mortality by the Medico-Sociological Committee of the British Medical Association. This Committee was appointed experimentally by the Council of the Association last autumn to consider social and economic questions affecting the public welfare as to which the medical profession has special knowledge. It was further charged to take such steps as might be found necessary to create or develop public opinion thereon. The first subject to which the Committee, after discussion, directed its attention was Maternity and Child-Welfare Work, and the report just issued from the British Medical Association office (price 3d.) is the outcome of its deliberations and inquiries.

### Composition of the Committee.

The original Committee included the officers of the British Medical Association—*i.e.*, the President, the Chairman of the Council, the Treasurer, and the Chairman of Representative Meetings. Of these the first three were unable to attend the meetings and therefore do not sign the report. The remaining original members were Mr. E. B. Turner, Mr. N. Bishop Harman, Dr. H. B. Brackenbury, Chairmen respectively of the Medico-Political, Hospital, and Insurance Acts Committees, Dr. T. Ridley Bailey, Dr. E. Rowland Fothergill, Mr. A. Forbes, Dr. A. C. Farquharson, and Mr. S. Morton Mackenzie, who co-opted the following: Mrs. Ogilvie Gordon (National Council of Women of Great Britain and Ireland), Miss J. Halford (National League for Health, Maternity, and Child Welfare), Miss Olive Haydon (Loughborough Junction Maternity and Child-Welfare Centre and member of the Central Midwives Board), Mrs. H. B. Irving (National Baby Week Council), Mr. J. R. Kaye (nominated by County Councils Association), Mrs. Kent Parsons, Public Health Department, Tottenham (nominated by the Women Sanitary Inspectors and Health Visitors' Association), and Dr. Eric Pritchard. The Women's Co-operative Guild, though unable to send a representative to sit on the Committee, forwarded its views on some of the points raised. Dr. E. Rowland Fothergill was appointed chairman, and Dr. Alfred Cox, medical secretary of the British Medical Association, acted as secretary of the Committee. A questionnaire was circulated and evidence received from various bodies representing medical officers of health, medical women, midwives, voluntary associations for child welfare, and health visitors, and from individuals, including Dr. W. A. Brend, and Dr. Shackleton, a general practitioner in Bradford.

### Three Stages of Development.

Covering the ground generally accepted to be included in the expression "maternity and child welfare," the Committee traces the three stages of development of the work in this country—viz., from 1900 to 1914, the period of private effort and permissive legislation; from 1914 to 1918, encouragement by the Government of local effort; and since 1918 the great development of State aid and control. The two main factors on which the development of the work has depended have been the desire to ameliorate individual suffering and efforts to increase national efficiency. Just as the Great War stimulated these factors, so the Boer War, combined with the falling birth-rate, stimulated to increased activity the philanthropic agencies which were at work previous to 1900. The first milk depôt was founded at St. Helen's in 1889; the first Infant Consultation Centre was started by Dr. Eric Pritchard at Marylebone in 1906; and the first School for Mothers was founded at St. Pancras by Dr. J. F. Sykes, then its medical officer of health, in 1907. Mr. John Burns, then President of the Local Government Board, gave his sympathetic support to conferences on infant mortality which were organised by a voluntary committee in 1906, and in the following year the medical inspection of school-children was made obligatory and the Notification of Births Acts (permissive until 1915 when it was made compulsory) was passed. Beginning in 1908 too the Board of Education made annual grants for schools for mothers. Thus the seed was sown and the ground prepared for the greater development of maternity and child-welfare work, which followed the Local Government Board's action in issuing in 1914 conditions for the payment of exchequer grants in aid of both voluntary and public schemes for maternity and child welfare. In 1918 the Maternity and Child Welfare Act was passed giving local authorities power to make arrangements under sanction from the Local Government Board for attending to the health of nursing mothers and children up to five years of age. In July 1919 there were 1,550 such Centres in existence, of which 660 were voluntary; in 1920 the number had increased to 1,754, of which 693 were provided by voluntary associations, and on January 1, 1921, there were in England and Wales 1,923 centres, of which 735 were provided by voluntary associations. The total grant from Government sources rose from £56,809 in 1915-16 to £777,000 in 1920-21.

### The Official Policy.

The Committee sums up the policy of the Ministry of Health in relation to this work, as detailed in the annual report of the Ministry 1919-20, Part I. It includes the development of centres, arrangements for consultations and treatment, the appointment of specially qualified medical officers, preferably women, the employment of more health visitors with the improvement of their training, the extension of midwifery services, the provision of sufficient hospital accommodation for diseases of pregnancy, confinements, and illnesses of infants and young children, of maternity homes and home nursing, and the supply of adequate nourishment for expectant and nursing mothers.

It summarises the method of administration of such schemes under the Act, viz., by a special statutory committee of the local authority, and points out the fact that the Local Government Board circular of 1919 strongly recommended that voluntary workers and women should be included on the sub-committees administering individual centres. About one-third of the centres are conducted by voluntary committees. The tendency is for the work to be under the control of a part-time or whole-time medical officer, though some centres are staffed by local practitioners, a practice which the British Medical Association Representative Body recommended in 1916, but which has been rendered difficult of accomplishment owing principally to the rapid growth of the centres.

### Infant Mortality at Varying Age Periods.

Some interesting tables of the mortality rate of infants and the causes of death are given in the report. The principal figures must be well known to our readers who have followed this subject in our pages, but their interest is increased by an analysis of infant mortality per 1,000 births from 1898 to 1919, according to age periods, *i.e.*, under one month, two to three months, four to six months, and seven to twelve months. The decrease in the mortality in the last two periods is very marked, viz., from 35.2 and 50.1 in 1898 to 13 and 21 respectively in 1919; in the first period it is slight, *i.e.*, from 41.8 in 1905 (the earliest year given) to 40 in 1919, and in the second period from 24.8 to 15. Dr. W. A. Brend is of opinion that there is a non-preventable infant mortality of from 25 to 30 per 1,000, and that these deaths occur almost entirely in the first month after birth.

The report then proceeds to discuss how far the diminution of infant mortality can be ascribed to the work of the maternity and child-welfare centres. The Committee had the advantage of the opinion of persons with much practical experience, all of whom ascribe a considerable portion of the diminution to maternity and child-welfare work, using the term in its broadest sense to include not only the work done at the Centres but the auxiliary work, and the indirect educational influence brought to bear. The Centres, in fact, should be regarded as a developing part of the general work. It finds that the gratuitous supply of artificial food and dried milk has proved detrimental to the best interests and influence of the Centres. The report remarks that it is very difficult to compare the influences exercised by maternity and child-welfare work and by other health agencies respectively, and that it is too early yet to determine to what extent the reduction in infant mortality is due to maternity and child-welfare work, though it states this work has contributed to an appreciable extent to the improvement. There are also other causes to be taken into consideration in estimating results, viz., the greatly improved economic conditions of large sections of the population, recent favourable climatic conditions, and the increased attention that is being paid to the "racial poisons" of alcohol, syphilis, and tubercle.

### The Committee's Conclusions and Recommendations.

On the subject of management and staffing the Centres the Committee has much to say, and has evidently given earnest consideration to this matter. The thorny question of voluntary agencies versus official control is gone into, and the case for the support and conduct of the work under the auspices of the local authority is put forward. In regard to staffing, the Committee finds there is much to be said for the employment of women practitioners, but many men are both qualified for and interested in the work and should not be excluded. The report concludes with a series of recommendations, which may be summarised as follows:

(*a*) Educational work amongst the mothers should be encouraged and extended, as should also the instruction of the elder girls at school in homecraft and mothercraft; (*b*) the improvement of the economic position and prospects of midwives and midwifery nurses, with encouragement by local authorities to secure a sufficient supply; (*c*) the provision of sufficient hospital accommodation for diseases of pregnancy and difficult confinements and maternity homes for normal confinements; (*d*) the primary and main object of maternity and child-welfare centres should be educational, preventive, and advisory; no treatment should be given for conditions which, in the absence of the Centre, would be recognised as calling for the attendance of a medical practitioner; it is against the best interests of the Centres to encourage women to go to the Centre for what they can get rather than for what they can learn; (*e*) there are advantages to the work in associating with the Centres a body of voluntary workers, but the whole of the work should be under the control of the medical officer of health in order that all the preventive agencies may be co-ordinated; (*f*) the support of the local doctors, nurses, and midwives can and ought to be secured, which can be done by making clear the restriction of the sphere of the Centres and by the representation of members of these professions on the statutory committee and the committees controlling the Centres; (*g*) practitioners accepting appointments on the Centres must have knowledge of and real interest in the work and must undertake to carry on the work irrespective of other claims on their time; (*h*) for every mother and child up to five years of age there should be available domiciliary attendance by a family doctor; (*i*) in order that medical practitioners should be fully equipped for maternity and child-welfare work it is desirable that the medical student should be educated and encouraged in the practice of duties relating to personal and domestic hygiene, industrial hygiene, and other spheres of preventive medicine.

